# Review of Clinical Trials on the Effects of Tai Chi Practice on Primary Hypertension: The Current State of Study Design and Quality Control

**DOI:** 10.1155/2020/6637489

**Published:** 2020-12-30

**Authors:** Yuke Teng, Sha Yang, Yuan Chen, Yuyi Guo, Yushi Hu, Pan Zhang, Jingya Cao, Xinyue Zhang, Yalan Chen, Caili Jiang, Tianyu Liu, Fang Zeng

**Affiliations:** ^1^Acupuncture and Tuina School/The 3rd Teaching Hospital, Chengdu University of Traditional Chinese Medicine, Chengdu, Sichuan, China; ^2^International Education School, Chengdu University of Traditional Chinese Medicine, Chengdu, Sichuan, China; ^3^Sports Medicine and Health School, Chengdu Sport University, Chengdu, Sichuan, China; ^4^Sport Hospital Affiliated to Chengdu Sport University, Chengdu, Sichuan, China; ^5^Sports School, Chengdu University of Traditional Chinese Medicine, Chengdu, Sichuan, China

## Abstract

The modulation of Tai Chi in physiological function and psychological status attracts sustaining attention. This paper collected original articles regarding the effects of Tai Chi practice on modulating primary hypertension from 7 electronic databases (PubMed, Excerpta Medica Database, Cochrane Library, Web of Science, Chinese Knowledge Resource Integrated Database, Wanfang Database, and China Science and Technology Journal Database) from their dates of origin to October 1st, 2020. A total of 45 articles were included. The literature analyses have shown that the benefits of Tai Chi practice for blood pressure management have been identified in all of the included 45 studies, and Tai Chi exercise has shown significant efficacy in improving hypertension clinical symptoms and quality of life, compared to the majority of control interventions, though there are also some methodological issues, including small sample sizes, lack of exact randomization methods and quality control criteria, and lack of specific standards used to measure the characteristics of Tai Chi practice. In the future, the inclusion of additional design standards, stricter quality controls, and evaluation measures for the features of Tai Chi practice is required in trials evaluating its effects on hypertension.

## 1. Introduction

As a classical mind-body exercise originating from ancient China, Tai Chi has become an important health-preserving approach. It emphasizes the coordination of motor skills, breathing, and mental awareness to keep the body and environment in harmony in order to preserve health and prevent and treat diseases. Clinical trials and systematic reviews have shown that Tai Chi practice is beneficial for both healthy subjects and patients with chronic diseases. On the one hand, Tai Chi practice is effective for keeping the dynamic balance of physical and psychological states for healthy people [1, 2]. On the other hand, it is also effective for improving symptoms, health-related quality of life (QoL), and emotional disorders of patients with chronic diseases as well as people with suboptimal health [3–5].

After analyzing original papers published between 1990 to 2020 on the health and therapeutic effects of Tai Chi practice, it was found that most papers focused on the efficacies of Tai Chi practice for chronic diseases, such as hypertension, type 2 diabetes, and osteoarthropathy disorders. It is noteworthy that 45 original papers centered on the efficacy of Tai Chi for hypertension, and the top-ranked original papers on the topic of Tai Chi practice were those examining the relationship between Tai Chi practice and chronic diseases. Most of the original papers on Tai Chi practice for hypertension have proven its benefits for blood pressure (BP) management and QoL improvement.

In the 2020 International Society of Hypertension Practice Guidelines [6], hypertension was defined based upon the following criteria: (1) systolic blood pressure (SBP) ≥ 140 mmHg and/or diastolic blood pressure (DBP) ≥ 90 mmHg, for blood pressure (BP) measured while in the office; (2) SBP ≥ 135 mmHg and/or DBP ≥ 85 mmHg, for BP measured while at home; or SBP ≥ 130 mmHg and/or DBP ≥ 80 mmHg, for BP measured during 24-hour ambulatory monitoring; or SBP ≥ 135 mmHg and/or DBP ≥ 85 mmHg, for BP measured during the daytime; or SBP ≥ 120 mmHg and/or DBP ≥ 70 mmHg, for BP measured during the night. As a major risk factor of cardiovascular disease (CVD), hypertension significantly impacts QoL for patients and carries with it remarkable healthcare cost burdens [7], with estimated global prevalence ranges between 30–45% [7, 8]. Currently, pharmacotherapy is the main treatment option for hypertension, and the majority of hypertension patients use two or more drugs in combination in order to achieve the desired BP range [6, 8–10]. However, the therapeutic effects of pharmacotherapy is unsatisfactory [7, 10], and their side effects include increased risk of skin cancer [11], erectile dysfunction [12], electrolyte disturbances [13], and worsening glucose intolerances [14]. The importance of lifestyle measures for BP management has become gradually recognized, so regular exercising is recommended as a form of lifestyle therapy for combating hypertension by the International Society of Hypertension (ISH) [6], European Society of Cardiology/European Society of Hypertension (ESC/ESH) [8], and American College of Cardiology/American Heart Association (ACC/AHA) [9]. However, Tai Chi practice has not yet been recommended for BP management due to limited amounts of clinical evidence.

After searching the original articles that are available to date, this paper investigated the research status of Tai Chi practice for hypertension and analyzed specific methodological issues, including issues with study designs, protocols of Tai Chi exercise, and its quality control criteria, in order to provide references for future studies and promote applications of Tai Chi practice for chronic disease.

## 2. Methods

### 2.1. Search Strategy

**Figure 1:**
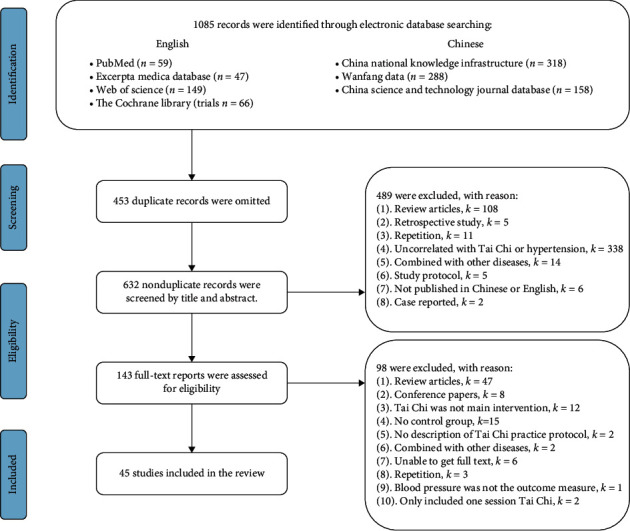
Flow chart detailing the systematic search of potential studies and selection process of included Tai Chi trials.

Data research was conducted in four electronic databases in English (PubMed, Excerpta Medica Database, Cochrane Library, and Web of Science) and three electronic databases in Chinese (Chinese Knowledge Resource Integrated Database, Wanfang Database, and China Science and Technology Journal Database). The publication time range was set from date of origin to October 1st, 2020. The full search strategy for each electronic database was listed in Supplementary Table 1. Then, screenings of the titles and abstracts were performed to identify eligible records. After screening, the full-text assessment was conducted according to the inclusion and exclusion criteria (Figure 1).

### 2.2. Inclusion Criteria

The studies were eligible if (1) participants were adults who were at least 18 years of age; (2) Tai Chi practice was explicitly established as the main or only intervention therapy other than control therapy; (3) the changes in BP from baseline to the end of intervention were reported in both Tai Chi and control groups.

### 2.3. Exclusion Criteria

The studies were excluded if (1) they were reviews, protocols, editorials, letters, or other studies of nonoriginal papers; or (2) there was no control group in the trials; or (3) there was no description of Tai Chi exercise protocol; or (4) the study only investigated the immediate effects resulting from a single session of Tai Chi exercise; or (5) subjects with chronic diseases (e.g., cardiovascular disease, diabetes mellitus, and cancer) were included in studies.

### 2.4. Data Extraction and Analysis

The data were extracted with regards to the following categories: (1) basic information including publishing time, title, author, language, and country of authorship; (2) information on study design including participants, sample size, randomization, control, and outcome measures; (3) protocol of Tai Chi exercise including the style, period, frequency, and number of sessions; (4) results of the study; (5) quality control criteria. Any inconsistencies or questions about data extraction were discussed and reconsidered until consensus was reached in our group. Data analysis was performed after data extraction.

## 3. Results

### 3.1. Basic Information

**Figure 2:**
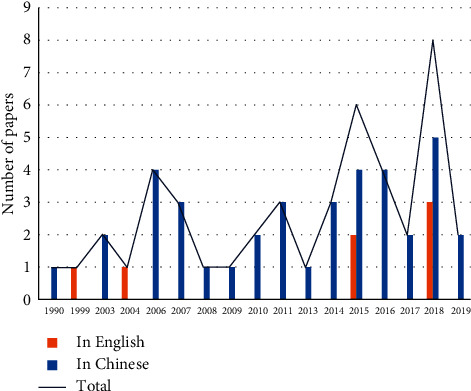
Annual and published quantity distribution of the 45 included studies.

A total of 45 studies were eventually included, numbered from S1 to S45. The basic information about them is listed in Supplementary Table 2. The first study of Tai Chi on hypertension was published in 1990 ^[S1]^ and peaked in 2018, when eight papers were published in the same year ^[S36–S43]^ (Figure 2). Among them, 44 studies were conducted in China (including one in Taiwan ^[S4]^ and one in Hong Kong ^[S40]^), and one study was conducted in the United States^[S2]^. The academic disciplines of authors mainly included physical education, medicine, and nursing professions.

### 3.2. The Study Design

The study design details and results of the 45 included studies are listed in Supplementary Table 3.

### 3.3. Participants

Among the 45 studies, forty-two studies recruited only hypertension patients ^[S1–S12, S14–S20, S22–S26, S28–S45]^, while the remaining 3 studies recruited both hypertension patients and healthy subjects ^[S13, S21, S27]^. The 31 studies explicitly described the hypertension grade which have enrolled the mild and moderate hypertension patients ^[S1–S5, S7, S10–S18, S21–S22, S24, S26–S28, S30, S32–S35, S37, S39, S42–S43, S45]^. Of the 31 studies, four studies also included patients with severe hypertension ^[S3, S7, S11, S13]^.

### 3.4. Randomization

Among the 45 studies, thirty-two mentioned randomizations as part of their methodologies ^[S2, S4, S6–S8, S12, S14–S17, S20–S23, S25, S28–S35, S37–S45]^. However, only 11 studies explicitly described their randomization methods ^[S6, S15, S25, S31–S32, S34, S38–S40, S43–S44]^, including simple random methods (nine studies in total ^[S25, S31, S32, S34, S38–S40, S43–S44]^, of which seven studies ^[S31, S32, S34, S38^^–^^S39, S43–S44]^ used a random number table and two studies ^[S25, S40]^, computer random method) and stratified random methods (two studies ^[S6, S15]^).

### 3.5. Sample Size

The sample sizes of 35 studies were equal to or less than 100 cases ^[S1–S9, S11, S13–S18, S20–S28, S31–S34, S37–S39, S43–S45]^, while ten studies were designed to have more than 100 cases ^[S10, S12, S19, S29–S30, S35–S36, S40–S42]^. The average sample size was 43 cases per group.

### 3.6. Control Group

**Figure 3:**
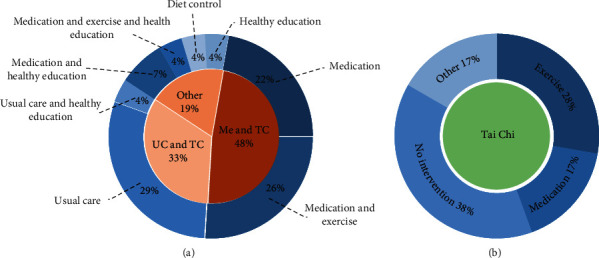
The control group aggregations of the 45 studies: (a) chart A lists the 27 studies that combined Tai Chi practice with other therapies as intervention groups; (b) chart B lists the 18 studies with Tai Chi exercise as the only intervention group. Me denotes medication; TC denotes Tai Chi; UC denotes usual care.

Within our study population of 45 studies, twenty-seven studies had participants engage in Tai Chi practice combined with other therapies, including medication, jogging, health education, usual care, and diet control as the intervention groups ^[S5–S10, S12, S14–S15, S18–S20, S25–S27, S31, S33–S37, S39, S41–S45]^. Within twenty-seven studies, eight studies established a usual care intervention group ^[S15, S20, S25, S27, S31, S41, S42, S44]^, six studies established a medication treatment intervention group ^[S6–S8, S18, S36, S39]^, seven studies set up a medication and exercise intervention group ^[S5, S9–S10, S14, S26, S37, S43]^, two studies established a medication and health education intervention group ^[S33, S35]^. Furthermore, a medication and exercise and health education intervention group ^[S34]^, a usual care and exercise intervention group ^[S45]^, a health education intervention group ^[S19]^, and a diet control intervention group ^[S12]^ were established as the control group in four studies, respectively (Figure 3(a)).

Eighteen studies established Tai Chi practice as the only intervention therapy ^[S1–S4, S11, S13, S16–S17, S21–S24, S28–S30, S32, S38, S40]^. Within these studies, seven studies only established a waiting list as the control group ^[S4, S16–S17, S21, S23, S24, S28]^, five studies established participants engaging in other exercises as various control groups ^[S2, S3, S11, S38, S40]^, three studies established participants taking medication as various control groups ^[S1, S30, S32]^, and three studies used other intervention methods as control groups ^[S13, S22, S29]^ (Figure 3(b)).

### 3.7. The Protocol of Tai Chi Exercise

#### 3.7.1. Style of Tai Chi

**Figure 4:**
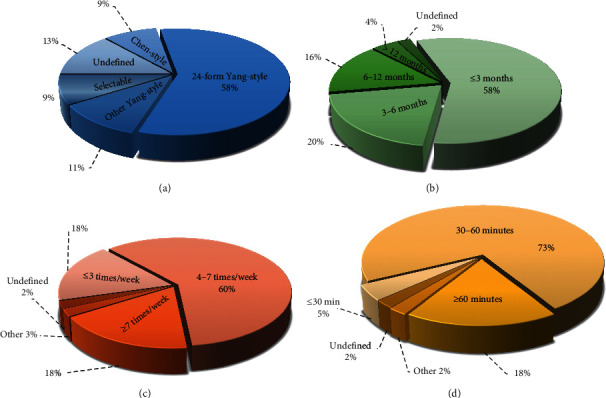
The protocol of Tai Chi exercise in the 45 studies: (a) for the style of Tai Chi; (b) for the intervention period; (c) for the exercise frequency; (d) for the length of each session.

Within our study population, 35 studies selected Yang-style Tai Chi as intervention therapy ^[S1–S6, S8–S10, S14–S18, S20–S24, S26–S28, S31–S34, S37–S45]^, and four studies selected Chen-style Tai Chi ^[S7, S12, S30, S35]^, while the other 6 studies did not report which Tai Chi styles were chosen ^[S11, S13, S19, S25, S29, S36]^. Among the 35 studies which selected Yang-style Tai Chi, 26 studies chose 24-form Tai Chi ^[S3, S6, S8–S9, S15–S17, S20–S24, S26–S27, S31–S34, S37, S39–S45]^, one study chose eight-form Tai Chi ^[S38]^, one study chose 13-form Tai Chi ^[S2]^, one study chose 48-form Tai Chi ^[S10]^, one study chose 108-form Tai Chi ^[S4]^, one study chose Jiang Ya Gong Tai Chi ^[S28]^, and four studies allowed the participants to freely choose the form of Tai Chi according to their preference ^[S1, S5, S14, S18]^ (Figure 4(a)).

#### 3.7.2. Intervention Period

The intervention period was designed to be equal to or less than three months for 26 studies ^[S2, S4, S6–S7, S9, S12–S13, S15–S17, S20–S24, S27, S30–S34, S38, S42–S45]^, more than three months and equal to or less than six months for nine studies ^[S5, S8, S10, S14, S18, S28, S35, S37, S41]^, more than six months and equal to or less than 12 months for seven studies ^[S1, S3, S11, S25, S29, S36, S40]^, and more than 12 months for two studies ^[S19, S39]^. One study did not mention the intervention period ^[S26]^ (Figure 4(b)).

#### 3.7.3. Exercise Frequency

The intervention frequency of Tai Chi was designed to be equal to or less than three times per week for 8 studies ^[S4, S11, S17, S28, S34, S37, S43, S44]^, four to seven times (including seven times) per week for 27 studies ^[S2–S3, S6–S9, S12–S14, S16, S18–S22, S24–S27, S32–S33, S36, S38–S41, S45]^, and more than seven times per week for 8 studies ^[S1, S10, S15, S23, S30–S31, S35, S42]^. In one study, participants were free to choose either of the two intervention frequencies based on their preference (twice per day, or once every other day) ^[S5]^. In addition, one study did not mention the intervention frequency ^[S29]^ (Figure 4(c)).

#### 3.7.4. Length of Each Session

Two studies designated less than 30 minutes for each session ^[S23, S31]^. Thirty-three studies designated session lengths ranging from 30 to 60 minutes ^[S1–S2, S4–S8, S10–S14, S18–S21, S24–S28, S30, S32–S35, S38–S41, S43–S45]^, and eight studies designated more than 60 minutes for each session ^[S3, S9, S16–S17, S22, S36–S37, S42]^. One study reported a slightly different design, requiring participants to practice in the group setting for three hours per week and then to practice individually for two hours per week at home^[S29]^, and one study did not report the length of time for each session^[S15]^ (Figure 4(d)).

### 3.8. Study Results

#### 3.8.1. Outcome Measures

All 45 studies selected BP changes as the measure of primary outcome. Additionally, the blood glucose levels, QoL, body mass index, serum nitric oxide levels, serum endothelin levels, blood lipid levels, total cholesterol, and waist circumference measures were also used to evaluate the efficacy of Tai Chi practice for hypertension.

#### 3.8.2. Study Results

All 45 studies reported that Tai Chi exercise is effective in combating hypertension symptom and/or increasing QoL, when compared to baseline measures. Compared with control groups, thirty eight of the 45 studies demonstrated the superiority of Tai Chi practices on its own, or of combining Tai Chi practices with other therapies for BP management ^[S1, S3–S12, S15–S17, S20–S29, S31, S33–S45]^. One study reported that the efficacy of combining Tai Chi practices with medication for hypertension was less effective than combining Qigong practice with medication ^[S14]^. Five studies ^[S2, S18, S19, S30, S32]^ reported that Tai Chi practice was similar to other therapies intended to regulate BP, including three studies comparing Tai Chi practice to medication ^[S18, S30, S32]^, one study comparing Tai Chi practice to moderate aerobic exercise ^[S2]^, and one study comparing Tai Chi practice to health education ^[S19]^. One study demonstrated that after Tai Chi exercise, the BP of the hypertension patients tended towards that of healthy subjects ^[S13]^.

#### 3.8.3. Adverse Reactions

No adverse reactions or side effects of Tai Chi practice were reported in the 45 studies.

## 4. Discussion

Tai Chi practice has been widely accepted as an effective approach for health care [15], an adjunctive treatment for chronic disorders [16], and a form of rehabilitation therapy during the recovery period from diseases [17, 18]. In the past three decades, the modulation of Tai Chi practice towards physiological function and psychological state for chronic diseases attracted global attention, especially with regards to primary hypertension. Hundreds of studies on the efficacies of Tai Chi practices for chronic diseases were conducted in more than a hundred institutions spread across 24 countries. However, the methodologies of these studies vary drastically, especially in terms of the design of Tai Chi exercise schemes and in terms of the evaluation of exercise efficacies, which in turn affects the objective evaluation in determining the efficacy of Tai Chi towards chronic diseases, as well as its clinical applications. Therefore, based on the original articles published in English and Chinese, this study aimed to summarize and analyze the data on the efficacies of Tai Chi practices for primary hypertension by examining the study design, protocols, results, and quality control criteria for Tai Chi practices, so as to provide a foundation for further studies and to promote the application of Tai Chi practice for chronic diseases, including hypertension.

### 4.1. The Study Design of Tai Chi for Hypertension

#### 4.1.1. Participant Inclusion

Grade I or grade II hypertension patients were involved in all 45 studies, and only four studies included grade III patients. In fact, mild and/or moderate patients were included in the majority of studies of Tai Chi on chronic diseases [19, 20] due to its modulating and low-risk characteristics.

Due to its relatively limited therapeutic effects, Tai Chi is usually used as a complementary therapy for chronic disease. Despite being relatively low-risk, sometimes Tai Chi practice may lead to falls or subsequent injuries, especially for the elderly and for those in poor physical condition. Consequently, the participants of Tai Chi studies were predominantly patients suffering from mild and moderate grades of hypertension. In the future, a possible direction of study may be on the demographic population that is best suited to practicing Tai Chi so as to maximize its applications in BP control.

#### 4.1.2. Sample Sizes and Randomization

The analysis showed that a total of 4236 subjects were included in the 45 studies. The minimum single-group sample size was 10, and the maximum was 238. In general, the sample sizes of these studies were relatively small. Among these studies, 32 studies mentioned randomization, while only 11 studies explicitly described the method of randomization. The small samples and lack of rigorous randomization are possible causes for poor quality and repeatability of the studies [21, 22]. Future studies should fully take these factors into consideration.

#### 4.1.3. Controls

Evaluating the therapeutic effects and investigating the advantages of Tai Chi practices for hypertension were the main purposes of these studies. For example, twenty-four studies ^[S1, S6–S8, S12–S13, S15, S18–S20, S22, S25, S27, S29–S33, S35, S36, S39, S41–S42, S44]^ aimed to verify the effectiveness of Tai Chi practice intervention by comparing it to positive drug or nondrug therapies, and fourteen studies ^[S2, S3, S5, S9–S11, S14, S26, S34, S37, S38, S40, S43, S45]^ were performed to investigate the advantages of mind-body modulation of Tai Chi over the other exercises. In the future, the control groups can be set up according to the following aspects so as to investigate the characteristics of Tai Chi for hypertension: firstly, Tai Chi practice can be compared with psychological intervention or exercise alone in order to observe the integrative effects of Tai Chi on BP control along with meditation or aerobic exercise. The effects of meditation, as a classical psychological method of intervention, on stress relief and BP reduction have been evaluated and confirmed in the past [23]. Furthermore, aerobic exercise is the primary exercise modality that professional organizations throughout the world recommend in terms of preventing and treating hypertension [6, 8, 9]. Therefore, since Tai Chi is a commonly used form of mind-body exercise, exploring the differences between effects of Tai Chi, psychotherapy, and aerobic exercise in hypertension control may yield great value. In the future, it may be possible to design clinical studies comparing Tai Chi with meditation or aerobic exercise in the intervention of hypertension. Secondly, both hypertension and hypotension patients can be included simultaneously to investigate the bidirectional effects of Tai Chi practices on BP modulation. Thirdly, both patients with hypertension (pathological state) and healthy subjects (physiological condition) can be included simultaneously to observe the benign modulating effects of Tai Chi practices on BP regulation.

#### 4.1.4. Selection of Outcome Measures

In most studies, the BP value was used as the main indicator, along with blood biochemical indices (serum glucose, blood lipid, cholesterol, nitric oxide endothelin levels, etc.) and biometric factors (body mass index and waist circumference). As the 2020 International Society of Hypertension Global Hypertension Practice Guidelines [6] suggested, the treatment of hypertension does not only focus on controlling BP but also emphasizes its prevention and reduction of risk factors and overall cardiovascular risk. So other than BP, these blood biochemical indices and biometric factors are also used to evaluate the effects of Tai Chi practice for hypertension. Furthermore, because Tai Chi is a type of mind-body exercise, some studies selected emotional scores, psychological scores, or QoL scores as outcome measures. Examples included the QoL Short Form 36-Item Health Survey, World Health Organization Quality of Life Brief Version, Pittsburgh Sleep Quality Index, Self-Rating Anxiety Scale, Self-Rating Depression Scale, and Geriatric Depression Scale. These indicate that the efficacy assessment of Tai Chi consists of multidimensional evaluations [24] due to its overall adjustment function. This feature can be found in almost all clinical studies on Tai Chi [25, 26]. However, we found that there is a lack of specific scale to reflect the physical and mental regulation of Tai Chi. Therefore, it will be necessary to establish specific scale of Tai Chi based on its characteristics.

### 4.2. Protocol of Tai Chi Exercise

The protocol of Tai Chi exercise can be analyzed in terms of four aspects: the style of Tai Chi, the period and the frequency of exercise, and the length of each session.

#### 4.2.1. Style and Form of Tai Chi

At present, Tai Chi styles mainly include the Yang-style, Chen-style, Wu-style, and Sun-style. In the 45 studies analyzed, 35 studies (78%) chose Yang-style, 4 studies (9%) chose Chen-style, and 6 studies (13%) did not specify the exact style.

Yang-style Tai Chi has the characteristics of smooth rhythms and gentle movements and requires a moderate volume of exercise, which renders it easy to practice. Comparatively, Chen-style Tai Chi is characterized by alternating rhythms, complex movements, larger exercise volume requirements, and higher demands on the physical condition. Therefore, most of the studies and clinical trials chose Yang-style Tai Chi as intervention [27–29].

Yang-style Tai Chi consists of 8-form, 13-form, 24-form, 42-form, 48-form, and 108-form. Within the 35 studies of Yang-style Tai Chi, 74% of these selected the 24-form for the following reasons: Firstly, the 24-form Yang-style Tai Chi was established and published in the form of a standardized exercise video by the General Sports Administration of China; secondly, Yang-style Tai Chi has a large practice following worldwide due to its high popularity and ease-of-acceptance [30]; lastly, the difficulty of 24-form Yang-style Tai Chi is moderate and it is easy to master. In the 45 studies analyzed, the average age of participants is around 60 years old. When they practiced Tai Chi, the complex motions would increase the learning curve and increase the risk of falls, strains, and sprains. Therefore, the 24-form Yang-style Tai Chi was most commonly selected in these studies. It is believed that 24-form Yang-style Tai Chi would be the best choice in future studies, evaluating the therapeutic effects of Tai Chi, except when investigating the differences in different styles/forms of Tai Chi.

#### 4.2.2. Exercise Volume of Tai Chi

The exercise volume of Tai Chi is primarily decided by three factors: the intervention period, exercise frequency, and length of each session. The volume of exercise is mainly based on its effectiveness and feasibility [31–33].

In 45 studies, the longest intervention period was two years, while the shortest was six weeks. The majority periods of these studies were equal to or less than three months. For the exercise frequency, eight studies (18%) reported exercise frequencies equal to or less than three times per week, twenty-seven studies (60%) reported exercise frequencies that were between four to seven times per week, eight studies (18%) reported exercise frequencies that were more than seven times per week, and one study (2%) allowed participants to freely select one of the two intervention frequencies based on their preference (twice per day or once every other day), while one study (2%) did not report the exercise frequency. In summary, the periods of equal to or less than three months and the fixed frequency of between four to seven times per week were the most commonly selected in these studies.

It is noteworthy that other than fixed exercise frequencies, some studies designated variable frequencies. For example, in one study investigating effects of Tai Chi exercise on arteriosclerosis [34], the variable frequency was established as once a week for the first three months and twice a week for the following nine months. In another study involving lower back pain [35], the variable frequency was designated as twice per week for eight weeks and once per week for the remaining two weeks. Among the 45 included studies, the length of each session ranged from 30 to 60 minutes in 33 studies (73%), to more than 60 minutes in 8 studies (18%), to less than 30 minutes in only 2 studies (5%). This indicates that moderate exercise volume may be more suitable for patients with hypertension. However, only nineteen studies described the specific time distribution, such as stretching and relaxing for 5–15 minutes and then practicing Tai Chi for 30–50 minutes. Based on the different exercise guidelines for fitness-improving activities, a 60-minute exercise is beneficial for muscular recovery [36] and risk reduction of motor impairment [37]. Therefore, it is recommended that each session be around 60 minutes, with 20 minutes of warm-up exercises and 30–40 minutes of Tai Chi exercises.

### 4.3. Quality Control

It is known that stricter quality control is key to improving the reliability and reproducibility of studies. In the 45 studies analyzed, 23 studies (51%) mentioned quality control criteria, including evaluation of Tai Chi teaching/training and exercise intensity. In fact, the normative movements of exercise are closely related to its therapeutic effects [38–40]. Therefore, it is necessary to prepare a detailed Tai Chi teaching protocol before formal study to maintain the quality of Tai Chi exercise. With regards to exercise intensity evaluation, 20 studies (44%) had mentioned some form of exercise intensity evaluation. It is essential to analyze and evaluate the body condition, especially in cases where patients are suffering from severe hypertension, for more elderly participants, in cases where a complex style/form of Tai Chi was selected, or in cases with large exercise volume. Most of the studies established heart rate or oxygen uptake as exercise intensity evaluation metrics. Furthermore, it should be noted that Tai Chi, as a classical mind-body exercise, requires the coordination of motor skills, breathing, and mental awareness in order to maintain the quality of exercise. However, specific methods of evaluating the coordination of motor skills, breathing, and mental awareness in Tai Chi exercise remain unclear. With the popularization of Tai Chi and the extensive number of Tai Chi studies being conducted globally, it is essential to develop relevant benchmarks and guidelines to evaluate the physical and mental effects of Tai Chi in a scientific manner.

In conclusion, Tai Chi practice for primary hypertension has been highlighted for its therapeutic values over the past 30 years. Reviewing these studies not only helps us to grasp the research status of Tai Chi practices for hypertension but also helps us to recognize the methodological issues to design better studies in the future. In the future, the benefits of Tai Chi practices for BP control should be further verified by multicenter and large sample RCTs, and the characteristics of Tai Chi practices for BP modulation should be clarified by investigating its integrity and bidirectional and benign regulation; a proper evaluative scale in accordance with the features of Tai Chi also needs to be developed, and the therapeutic mechanism of Tai Chi also needs to be explored.

## Data Availability

The information of the included 45 studies used to support the findings of this study are included within the supplementary information files.

## Supplementary Materials

Supplementary Materials Supplementary Table 1: the full search strategy for each of the electronic databases queried, including the electronic databases name, vendor/platform, coverage, searches strategy, and hits number. Supplementary Table 2: the basic information of the 45 included studies, including study number, paper title, author name, author major, publish time, paper language, and author perform place. Supplementary Table 3: the study design, intervention protocol, and results of the 45 included studies, including the study design (participants, whether random, sample size, hypertension grade, intervention group, and control group), intervention protocol (Tai Chi-style, period, frequency, and time), and result (outcome measure and conclusion).
