# Supplementary material for: Review of Clinical Trials on the Effects of Tai Chi Practice on Primary Hypertension: The Current State of Study Design and Quality Control
Source: Evid Based Complement Alternat Med. 2020 Dec 30;2020:6637489. doi: 10.1155/2020/6637489 (PMC7787759; doi:10.1155/2020/6637489)
Supplement: Supplementary Materials — Supplementary Table 1: the full search strategy for each of the electronic databases queried, including the electronic databases name, vendor/platform, coverage, searches strategy, and hits number. Supplementary Table 2: the basic information of the 45 included studies, including study number, paper title, author name, author major, publish time, paper language, and author perform place. Supplementary Table 3: the study design, intervention protocol, and results of the 45 included studies, including the study design (participants, whether random, sample size, hypertension grade, intervention group, and control group), intervention protocol (Tai Chi-style, period, frequency, and time), and result (outcome measure and conclusion). [file 6637489.f1.zip › 6637489.f1/12.11-Supplementary Table 1.pdf]

Supplementary Table 1.Full search strategy for each of the electronic databases queried.

Electronic databases	Vendor/Platform	Coverage	Searches strategy	Hits
PubMed (including MEDLINE)	National Library of Medicine	Date of inception 1946 – October 1st, 2020	(“Tai Ji" OR” “Tai Chi” [MeSH Terms]OR “ Tai Ji Quan” OR "”T'ai Chi” OR “ Tai Chi Chuan” OR “ Tai-ji”) AND (“hypertension” [MeSH Terms] OR “primary hypertension” OR “Essential hypertension” OR “High Blood Pressure*” OR “Blood Pressure*, High” OR “pre hypertension” OR “borderline hypertension”)	59
Excerpta Medica Database	Elsevier Sci Verse	Date of inception 1974 – October 1st, 2020	Search #1:<br>“hypertension” OR “primary hypertension” OR “Essential hypertension” OR “High Blood Pressure” OR “Blood Pressure, High” OR “pre hypertension” OR “borderline hypertension”<br>Search #2:<br>“Tai Ji” OR “Tai Chi” OR “Tai Ji Quan” OR “T'ai Chi” OR “Tai Chi Chuan” OR “Tai-ji”<br>Then under search history: combined search #1 and #2	47
The Cochrane Library	Wiley Online Library	Date of inception 1999 – October 1st, 2020	Search<br>#1:“hypertension” #2: “primary hypertension”<br>#3:“Essential hypertension” #4: “High Blood Pressure”	Trials 66

			#5: “Blood Pressure, High” #6: “ Blood Pressures, High”<br>#7: “High Blood Pressures” #8: “pre hypertension”<br>#9: “borderline hypertension”<br>#10: #1 OR #2 OR #3 OR #4 OR #5 OR #6 OR #7 OR #8 OR #9<br>#11“ Tai Chi” #12 “ Taijiquan” #13 “Tai Ji Quan” #14 “Taiji”<br>#15 “Ji Quan, Tai” #16 “Chi, Tai” #17 “Tai Chi Chuan”<br>#18 “Quan, Tai Ji” #19 “Tai-ji” #20 “T'ai Chi”<br>#21:#11 OR #12 OR #13 OR #14 OR #15 OR #16 OR #17 OR #18 OR #19 OR #20<br>#22: #10 AND #21<br>Trials	
Web of Science	Clarivate Analytics	Date of inception<br>1950 – October 1st, 2020	Search #1:<br>“hypertension” OR “primary hypertension” OR “Essential hypertension” OR “High Blood Pressure” OR “Blood Pressure, High” OR “pre hypertension” OR “borderline hypertension”<br>Search #2:<br>“Tai Ji” OR “Tai Chi” OR “Tai Ji Quan” OR “T'ai Chi” OR “Tai Chi Chuan” OR “Tai-ji”	149

		Then under search history: combined search #1 and #2	
Chinese Knowledge Resource Integrated Database	Date of inception 1999 – October 1st, 2020	Search #1 (“高血压”+ “原发性高血压”+“高血压病”+“高血压患者”)<br>Search #2 (“太极” + “太极拳” + “太极拳运动” +“太极拳锻炼”)<br>#1 AND #2	318
Wanfang Database	Date of inception 1950 – October 1st, 2020	Search #1 (“高血压”+ “原发性高血压”+ “高血压病”+“高血压患者”)<br>Search #2 (“太极”+ “太极拳”+“太极拳运动”+“太极拳锻炼”)<br>#1 AND #2	288
China Science and Technology Journal Database	Date of inception 1999 – October 1st, 2020	Search #1 (“高血压”+ “原发性高血压”+ “高血压病” +“高血压患者”)<br>Search #2 (“太极”+ “太极拳”+“太极拳运动”+“太极拳锻炼”)<br>#1 AND #2	158
