# Supplementary material for: Review of Clinical Trials on the Effects of Tai Chi Practice on Primary Hypertension: The Current State of Study Design and Quality Control
Source: Evid Based Complement Alternat Med. 2020 Dec 30;2020:6637489. doi: 10.1155/2020/6637489 (PMC7787759; doi:10.1155/2020/6637489)
Supplement: Supplementary Materials — Supplementary Table 1: the full search strategy for each of the electronic databases queried, including the electronic databases name, vendor/platform, coverage, searches strategy, and hits number. Supplementary Table 2: the basic information of the 45 included studies, including study number, paper title, author name, author major, publish time, paper language, and author perform place. Supplementary Table 3: the study design, intervention protocol, and results of the 45 included studies, including the study design (participants, whether random, sample size, hypertension grade, intervention group, and control group), intervention protocol (Tai Chi-style, period, frequency, and time), and result (outcome measure and conclusion). [file 6637489.f1.zip › 6637489.f1/12.11-Supplementary Table 2.pdf]

Supplementary Table 2.The basic information of the 45 included studies.

Study No.	Title	Author Name	Author Major	Publish Time	Paper Language	Perform Place
S1	太极拳对高血压病人的防治作用	Yin J.et al.	/	1990	Chinese	China
S2	The Effects of Aerobic Exercise and T'ai Chi on Blood Pressure in Older People: Results of a Randomized Trial	Deborah RY.et al.	Medicine	1999	English	USA
S3	不同锻炼方式对老年人高血压疗效的研究	Wei Y.et al.	Physical Education	2003	Chinese	China
S4	The Beneficial Effects of Tai Chi Chuan on Blood Pressure and Lipid Profile and Anxiety Status in a Randomized Controlled Trial	Tsai JC.et al.	Nursing	2003	English	China
S5	Experimental Research on Shadowboxing Prescription of Middle, Old-aged Hypertensive	Chen YP.et al.	Physical Education	2004	Chinese	China
S6	Effect of Tai Chi exercise on blood pressure, plasma nitrogen monoxidum and endothelin in hypertensive patients	Mao HN.et al.	Physical Education	2006	Chinese	China
S7	Effects of Taijiquan Exercise on Hypertension Patients' NO Consistency in Plasma, the Activity of RBC Na+-K+ ATPase and Ca^{2+}-Mg^{2+} ATPase	Chen XX.et al.	Physical Education	2006	Chinese	China
S8	太极拳配合药物治疗原发性高血压的临床研究	Luo H	Medicine	2006	Chinese	China
S9	太极拳运动对中老年人高血压的影响	Ge WZ.et al.	Physical Education	2006	Chinese	China
S10	Comparison of the effects between Taijiquan and walking exercises on the rehabilitation of hypertension symptoms	Wang C.et al.	Physical Education	2007	Chinese	China
S11	太极拳和健身操对老年人高血压疗效之研究	Wang Y	Physical Education	2007	Chinese	China
S12	Effects of Tai Ji Quan on Blood Pressure and Lipid Profile in Patients with Stage I Essential Hypertension	Zhou SW	Medicine	2007	Chinese	China

S13	太极拳对原发性高血压患者循环 NO/ET 平衡性的研究	Mao HN.et al.	Physical Education	2008	Chinese	China
S14	Effects of Traditional Sports on Clinical Symptom of Aged Intellectual Patients with Essential Hypertension	Tang QH	Physical Education	2009	Chinese	China
S15	The effect of shadow boxing exercise on the long-term quality of life in middle-aged and elderly patients with primary hypertension	Han QY.et al.	Nursing	2010	Chinese	China
S16	Research on the effect of middle-aging and elderly high blood pressure patient' quality of life by practice Taichi	Sun QQ	Physical Education	2010	Chinese	China
S17	Effects of Taichi Exercises of Various Intensities on Essential Hypertension	Wang XJ.et al.	Physical Education	2011	Chinese	China
S18	太极拳对原发性高血压患者干预效果研究	He JH.et al.	Physical Education	2011	Chinese	China
S19	Effect evaluation of Tai Chi on hypertension in Guangzhou Liurong Community	Chen JY.et al.	Medicine	2011	Chinese	China
S20	太极拳对高血压患者血压的影响	Chen ZF.et al.	Nursing	2013	Chinese	China
S21	Gaseous Signal Molecular Mechanism of Taijiquan's Intervention in Aged Essential Hypertension	Xie HJ.et al.	Physical Education	2014	Chinese	China
S22	太极拳运动对老年高血压的干预效果	Sun F.et al.	Physical Education	2014	Chinese	China
S23	The effect of Tai Chi Exercise on the elderly patients with hypertension	Huang RF.et al.	Physical Education	2014	Chinese	China
S24	太极拳运动对社区中老年高血压患者降压效果的观察及其降压机制的探讨	Lu W.et al.	Physical Education	2015	Chinese	China
S25	同伴太极拳练习对老年高血压患者血压控制的影响	Wei YH.et al.	Nursing	2015	Chinese	China
S26	慢跑结合太极拳对中老年高血压患者的疗效及机制	Qiu LW.et al.	Physical Education	2015	Chinese	China
S27	Effects of Tai Chi exercise on blood pressure and plasma levels of nitric oxide, carbon monoxide and hydrogen sulfide in real-world patients with essential hypertension	Pan XG.et al.	Physical Education	2015	English	China

S28	Use antihypertensive tai chi gong improve the effective of different TCM constitution in elderly patients with high blood pressure	Cui CL	Physical Education	2015	Chinese	China
S29	Community-Based Mind-Body Meditative Tai Chi Program and Its Effects on Improvement of Blood Pressure, Weight, Renal Function, Serum Lipoprotein, and Quality of Life in Chinese Adults With Hypertension	Sun J.et al.	Medicine	2015	English	China
S30	The influence of exercise treatment on type I hypertension of elders	Li CY.et al.	Physical Education	2016	Chinese	China
S31	24 式简化太极拳对高血压患者血压以及生活质量的影响	Xu H	Medicine	2016	Chinese	China
S32	Effects of Taijiquan on 24-hour Dynamic Blood Pressure and Vascular Function in Patients with Primary Mild Hypertension	Jin HL	Medicine	2016	Chinese	China
S33	The Research Of Blood Pressure Variability And Quality Of Life Effect On Patients With Liver-yang Hyperactivity Type Hypertension By Taijiquan	Liu WQ	Medicine	2016	Chinese	China
S34	A Clinical Study :Effect of 24 Movement Form of Tai Chi on Senile Patients with Essential Hypertension	Zhang DL	Medicine	2017	Chinese	China
S35	Study on Intervention of Essential Hypertension Group by Tai Chi Combined with Health Management	Liu J	Medicine	2017	Chinese	China
S36	太极拳运动对中老年女性高血压患者的影响	Song YB	Physical Education	2018	Chinese	China
S37	The effect of Tai Chi on sleep quality in elderly patients with essential hypertension	Yang XW	Medicine	2018	Chinese	China
S38	八式太极拳运动对原发性高血压患者血压水平、血管内皮功能及生活质量的影响	Xiao YK	Physical Education	2018	Chinese	China
S39	太极拳运动对老年高血压患者血压、血液流变学及远期生活质量的影响	Liu T.et al.	Physical Education	2018	Chinese	China

S40	Tai Chi Exercise is More Effective than Brisk Walking in Reducing Cardiovascular Disease Risk Factors among Adults with Hypertension: A Randomised Controlled Trial	Aileen Chan WK.et al.	Nursing	2018	English	China
S41	The impact of group-based Tai chi on health-status outcomes among community-dwelling older adults with hypertension	Ma CH.et al.	Nursing	2018	English	China
S42	Effect of t'ai chi Exercise on Hypertension in Youngand Middle-Aged In-Service Staff	Shou XL.et al.	Medicine	2018	English	China
S43	24 式太极拳对老年原发性高血压患者降压效果及血脂水平的临床疗效评价	Feng LJ.et al.	Medicine	2018	Chinese	China
S44	24 式简化太极拳对老年原发性高血压伴轻度焦虑状态的影响	Wang XB.et al.	Medicine	2019	Chinese	China
S45	A comparative study of the effects of Taijiquan and walking exercise on physical and mental health of middle-aged and elderly patients with hypertension	Lin SF.et al.	Physical Education	2019	Chinese	China
