# Supplementary material for: Review of Clinical Trials on the Effects of Tai Chi Practice on Primary Hypertension: The Current State of Study Design and Quality Control
Source: Evid Based Complement Alternat Med. 2020 Dec 30;2020:6637489. doi: 10.1155/2020/6637489 (PMC7787759; doi:10.1155/2020/6637489)
Supplement: Supplementary Materials — Supplementary Table 1: the full search strategy for each of the electronic databases queried, including the electronic databases name, vendor/platform, coverage, searches strategy, and hits number. Supplementary Table 2: the basic information of the 45 included studies, including study number, paper title, author name, author major, publish time, paper language, and author perform place. Supplementary Table 3: the study design, intervention protocol, and results of the 45 included studies, including the study design (participants, whether random, sample size, hypertension grade, intervention group, and control group), intervention protocol (Tai Chi-style, period, frequency, and time), and result (outcome measure and conclusion). [file 6637489.f1.zip › 6637489.f1/12.11-Supplementary Table 3.pdf]

Supplementary Table 3.The study design intervention protocol and results of the 45 included studies.

Study No.	Study Design						Intervention Protocol				Result	
	Participants	Random	Sample Size	Grade	Intervention Group	Control Group	Tai Chi Style	Period	Frequency	Time	Outcome Measure	Conclusion
S1	Patients	N	20	I\II	Tai Chi	Medication	Selectable(Chen / Yang style)	12m	2t/day	60min	BP/ Haemoglobinometer / Symptom / Pulse rate	Positive result and superior to medication
S2	Patients	Y	62	I	Tai Chi	Exercise（Moderate intensity aerobic exercise)	Yang style 13 form Tai Chi	3m	6~7t/w	30～60	BP	Positive result and equal to exercise
S3	Patients	N	46	I/II/III	Tai Chi	Exercise（walking/jogging）	Yang style 24 form Tai Chi	12m	5t/w	80min	BP	Positive result and superior to exercise
S4	Patients	Y	88	I	Tai Chi	No intervention	Yang style 108 form Tai Chi	3m	3t/w	50min	BP/Blood lipid/ Emotional state	Positive result and superior to no intervention
S5	Patients	N	100	High normal / I /II	Medication & Tai Chi	Medication & Exercise	Selectable(Yang style 24 form / 42 form/ 42 form sword)	5m	Selectable (30-45 min, 1-2t/day or 45-60 min, every other day)	30-60min	BP/Pulse	Positive result and superior to M&E
S6	Patients	Y	62	Undefined	Medication & Tai Chi	Medication	Yang style 24 form Tai Chi	2m	6t/w	60min	BP/NO/ET	Positive result and superior to medication
S7	Patients	Y	40	II/III	Medication & Tai Chi	Medication	Chen style Tai Chi	10w	7t/w	40min	BP/NO/Na^{+}-K^{+}ATPase/ Ca^{2+}-Mg^{2+}ATPase	Positive result and superior to medication
S8	Patients	Y	84	Undefined	Medication & Tai Chi	Medication	Yang style 24 form Tai Chi	6m	7t/w	45min	BP/Hemorheology	Positive result and superior to medication
S9	Patients	N	50	Undefined	Medication & Tai Chi	Medication & Exercise (walking/dance.etc.）	Yang style 24 form Tai Chi	2m	7t/w	90min	BP	Positive result and superior to M&E
S10	Patients	N	126	High normal /I/II	Medication & Tai Chi	Medication /Medication & Exercise (walking)	Yang style 48 form Tai Chi	6m	2t/day	60min	BP	Positive result and superior to M&E
S11	Patients	N	46	I/II/III	Tai Chi	Exercise(body building exercise)	Undefined	8m	3t/w	60min	BP	Positive result and superior to medication

S12	Patients	Y	120	I	Diet control &Tai Chi	Diet control	Chen style Tai Chi	3m	7t/w	60min	BP/Blood lipid/HR	Positive result and superior to diet control
S13	Patients & healthy subjects	N	85	I/II/III	Tai Chi	Healthy	Undefined	2m	5~6t/w	≥30min	BP/NO/ET	Positive result
S14	Patients	Y	48	I/II	Medication & Tai Chi	Medication /Medication & Exercise (Qigong)	Selectable (Yang style 24 form or/and 48 form Tai Chi)	6m	3~5t/w	30~60min	BP	Positive result and lower to M&E(Qigong)
S15	Patients	Y	60	I/II	Usual Care& Tai Chi	Usual care	Yang style 24 form Tai Chi	3m	1~2t/day	Undefined	BP/SF-36	Positive result and superior to usual care
S16	Patients	Y	80	I/II	Tai Chi	No intervention	Yang style 24 form Tai Chi	3m	6t/w	90min	BP/SF36/Height/Weight/Waistline/Hipline	Positive result and superior to no intervention
S17	Patients	Y	41	I/II	Tai Chi with moderate intensity/ low intensity	No intervention	Yang style 24 form Tai Chi	3m	3t/w	90min	BP/Height/Weight/Waistline/HR/RPE	Positive result and superior to no intervention
S18	Patients	N	49	I/II	Medication &Tai Chi	Medication	Selectable (Yang style 24/42/48 form Tai Chi)	5m	6 t/w	40-50min	BP/ Exercise stress test	Positive result and equal to medication
S19	Patients	N	441	Undefined	Health education & Tai Chi	Healthy education	Undefined	24m	5t/w	60min	BP	Positive result and equal to health education
S20	Patients	Y	68	Undefined	Usual care & Tai Chi	Usual care	Yang style 24 form Tai Chi	3m	6t/w	60min	BP	Positive result and superior to usual care
S21	Patients & healthy subjects	Y	75	I/II	Tai Chi	No intervention	Yang style 24 form Tai Chi	3m	5t/w	60min	BP/NO/H_2S	Positive result and superior to no intervention
S22	Patients	Y	90	I/II	Tai Chi	Healthy education	Yang style 24 form Tai Chi	2m	7t/w	120min	BP/SAS/SDS/E/NE	Positive result and superior to health education
S23	Patients	Y	50	Undefined	Tai Chi	No intervention	Yang style 24 form Tai Chi	2m	1-2t/day	20~30min	BP/BMI/Weight/GLU/Blood lipid/HR/SAS/SDS	Positive result and superior to no intervention

S24	Patients	N	25	I	Tai Chi	No intervention	Yang style 24 form Tai Chi	2m	6t/w	60min	BP/Height/Weight/Pulse Rate/NO/ET/TG/TC	Positive result and superior to no intervention
S25	Patients	Y	84	Undefined	Usual care & Tai Chi	Usual care	Undefined	12m	7t/w	30-45min	BP	Positive result and superior to usual care
S26	Patients	N	26	High normal /I/II	Medication & Jogging & Tai Chi	Medication & Exercise (jogging)	Yang style 24 form Tai Chi	Undefined	4t/w	40-60min	BP/ Weight/BMI/ WHR	Positive result and superior to M&E
S27	Patients & healthy subjects	N	56	I/II	Usual care & Tai Chi	Usual care/ No intervention	Yang style 24 form Tai Chi	3m	6t/w	60min	BP/GLU/TC/NO/CO	Positive result and superior to usual care
S28	Patients	Y	60	I/II	Tai Chi	No intervention	Yang style Tai Chi Jiangyagong	6m	3t/w	60min	BP	Positive result and superior to no intervention
S29	Patients	Y	300	Undefined	Tai Chi	Non-drug treatment(reading & learning computer software)	Undefined	12m	Undefined	Group-3h/w； Home-2h/w	BP/Blood lipid/GLU/Waistline/BMI/Quality of life	Positive result and superior to non-drug treatment
S30	Patients	Y	120	I	Tai Chi	Medication	Chen style Tai Chi	2m	2t/day	40min	BP	Positive result and equal to medication
S31	Patients	Y	60	Undefined	Usual care & Tai Chi	Usual care	Yang style 24 form Tai Chi	2m	2t/day	<30min	BP/Quality of life	Positive result and superior to usual care
S32	Patients	Y	54	I	Tai Chi	Medication	Yang style 24 form Tai Chi	6w	7t/w	40min	24hBP/ET/NO	Positive result and equal to medication
S33	Patients	Y	60	I /II	Medication & Healthy education& Tai Chi	Medication &Healthy education	Yang style 24 form Tai Chi	3m	5t/w	40min	24hBP/ET/NO/ Quality of life	Positive result and superior to M&H
S34	Patients	Y	82	High normal /I/II	Medication & Healthy education& Tai Chi	Medication & Healthy education& Exercise(walking)	Yang style 24 form Tai Chi	3m	3t/w	60min	BP/ BMI/WHR/ Blood lipid	Positive result and superior to M&H&E
S35	Patients	Y	157	I/II	Medication & Healthy education& Tai Chi	Medication & Healthy education	Chen style Tai Chi	6m	2t/day	40mīn	BP/TG	Positive result and superior to M & H

S36	Patients	N	154	Undefined	Medication & Tai Chi	Medication	Undefined	9m	7t/w	<120min	BP/SF-36	Positive result and superior to medication
S37	Patients	Y	78	High normal /I/II	Medication & Tai Chi	Medication & Exercise（jogging）	Yang style 24 form Tai Chi	4m	3t/w	65-75min	BP/Blood lipid /hsCRP /Quality of life/ GDS/ PSQI/TCD	Positive result and superior to M&E
S38	Patients	Y	84	Undefined	Tai Chi	Exercise	Yang style 8 form Tai Chi	3m	5t/w	50min	BP/GLU/NO/ET	Positive result and superior to exercise
S39	Patients	Y	70	I /II	Medication & Tai Chi	Medication	Yang style 24 form Tai Chi	18m	7t/w	40-60min	BP/ Hemorheology /SF-36	Positive result and superior to medication
S40	Patients	Y	246	Undefined	Tai Chi	Exercise（brisk walking）/Usual care	Yang style 24 form Tai Chi	Group-12w;Home-24w	Group-2t/w Home-5t/w	Group-60min Home-30min	BP	Positive result and superior to exercise
S41	Patients	Y	158	Undefined	Usual care & Tai Chi	Usual care	Yang style 24 form Tai Chi	6m	3-5t/w	60min	BP/BMI/ Waistline	Positive result and superior to usual care
S42	Patients	Y	208	I	Usual care & Tai Chi	Usual care	Yang style 24 form Tai Chi	3m	1-2t/day	40-90min	BP /HR/ Blood lipid /BMI	Positive result and superior to usual care
S43	Patients	Y	82	High normal /I/II	Medication & Tai Chi	Medication & Exercise（walking）	Yang style 24 form Tai Chi	3m	3t/w	60min	BP/ Blood lipid	Positive result and superior to M&E
S44	Patients	Y	100	Undefined	Usual care & Tai Chi	Usual care	Yang style 24 form Tai Chi	3m	3t/w	40-60 min	BP/SAS/ /WHOQOL-BREF	Positive result and superior to usual care
S45	Patients	Y	41	I/II	Usual care & Tai Chi	Usual care & Exercise（brisk walking）/ Usual care	Yang style 24 form Tai Chi	10w	5t/w	45-60min	BP/HR/Physical condition/ RPE/SAS/SDS	Positive result and superior to usual care &exercise
